# Research on potential biomarkers in hereditary hemorrhagic telangiectasia

**DOI:** 10.3389/fgene.2015.00115

**Published:** 2015-03-31

**Authors:** Luisa-María Botella, Virginia Albiñana, Luisa Ojeda-Fernandez, Lucia Recio-Poveda, Carmelo Bernabéu

**Affiliations:** ^1^Department of Cellular and Molecular Medicine, Centro de Investigaciones Biológicas, Consejo Superior de Investigaciones Cientificas, Madrid, Spain; ^2^Centro de Investigación Biomédica en Red de Enfermedades Raras, Madrid, Spain

**Keywords:** biomarkers, HHT, endoglin, ALK1, miRNA, TGF-β, VEGF, Ang-2

## Abstract

Hereditary hemorrhagic telangiectasia (HHT) is a genetically heterogeneous disorder, involving mutations in two predominant genes known as Endoglin (ENG; HHT1) and activin receptor-like kinase 1 (ACVRL1/ALK1; HHT2), as well as in some less frequent genes, such as MADH4/SMAD4 (JP-HHT) or BMP9/GDF2 (HHT5). The diagnosis of HHT patients currently remains at the clinical level, according to the “Curaçao criteria,” whereas the molecular diagnosis is used to confirm or rule out suspected HHT cases, especially when a well characterized index case is present in the family or in an isolated population. Unfortunately, many suspected patients do not present a clear HHT diagnosis or do not show pathogenic mutations in HHT genes, prompting the need to investigate additional biomarkers of the disease. Here, several HHT biomarkers and novel methodological approaches developed during the last years will be reviewed. On one hand, products detected in plasma or serum samples: soluble proteins (vascular endothelial growth factor, transforming growth factor β1, soluble endoglin, angiopoietin-2) and microRNA variants (miR-27a, miR-205, miR-210). On the other hand, differential HHT gene expression fingerprinting, next generation sequencing of a panel of genes involved in HHT, and infrared spectroscopy combined with artificial neural network patterns will also be reviewed. All these biomarkers might help to improve and refine HHT diagnosis by distinguishing from the non-HHT population.

## Introduction

Hereditary hemorrhagic telangiectasia (HHT; OMIM 187300), or Rendu–Osler–Weber syndrome, is an inherited autosomal dominant disease affecting 1 in 5,000 individuals as assessed in several human populations (Shovlin, 2010). The disease is characterized by abnormal vascular structures, which lead to epistaxis, telangiectases, and anemia as well as visceral arteriovenous malformations (AVMs) in the lung, brain, and liver. These may contribute to serious health outcomes, such as strokes, brain abscesses, and hemorrhages (Shovlin, 2010; McDonald et al., 2011).

HHT is a genetically heterogeneous disorder. The first genes identified as mutated in HHT patients, were *Endoglin* (*ENG*), located on chromosome 9q33-34, causing HHT1 (Fernández-Ruiz et al., 1993; McAllister et al., 1994) and *ACVRL1/ALK1* (activin receptor-like kinase 1), that causes HHT2 (Johnson et al., 1995, 1996). Over 80–85% of the HHT patients present mutations in either *ENG* or *ACVRL1* genes, while unidentified mutations in additional loci account for the remaining cases. These loci include the HHT3 locus on chromosome 5 or the HHT4 locus on chromosome 7, whose genes remain unidentified (Cole et al., 2005; Bayrak-Toydemir et al., 2006), along with mutations in the *MADH4/SMAD4* gene causing familial juvenile polyposis associated with HHT (Gallione et al., 2004). More recently, mutations in *BMP9/GDF2* were described as the cause of an HHT-like syndrome (Wooderchak-Donahue et al., 2013).

**FIGURE 1:**
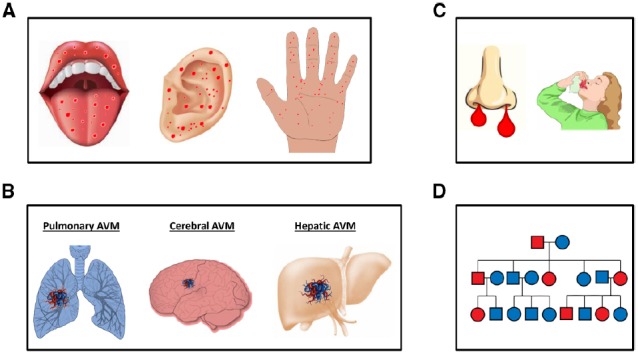
**Curaçao clinical criteria in HHT. (A)** Multiple telangiectases at characteristic sites, such as oral cavity, ears, and fingers. **(B)** Visceral lesions (AVMs) in the lungs, brain, liver or spinal cord, and gastrointestinal bleeding. **(C)** Epistaxis (spontaneous, recurrent nosebleeds). **(D)** An example of a family history of HHT; a genetic family tree with affected members in red and non-affected members in blue is shown.

The diagnosis of HHT currently remains at the clinical level. The consensus clinical criteria, known as “Curaçao criteria,” are epistaxis (spontaneous, recurrent nosebleeds); telangiectases (multiple at characteristic sites, such as the lips, oral cavity, fingers, and nose); visceral lesions (AVMs in the lung, brain, liver, or spinal cord), gastrointestinal bleeding, and a family history (Faughnan et al., 2011; Figure 1). Curaçao criteria are particularly helpful in identifying affected from non-affected adults. However, special attention must be paid to the risk of not diagnosing HHT in asymptomatic children and young adults. In these cases, not all of the typical symptoms may be present. In general, most patients show a full penetrance of the disease around the age of 40 (Shovlin, 2010; Faughnan et al., 2011).

The genetic testing for HHT genes is the choice option in children and teenagers, especially if the family mutation is known. In these cases, the genetic results should be definite for either the positive diagnosis or exclusion. Current genetic determinations imply the sequencing of at least two HHT genes. This process is time-consuming, expensive and in 10–15% of the cases, the mutation is not identified. Therefore, it would be desirable to have alternative tools facilitating diagnosis. They should ideally allow earlier, faster, cheaper, and easier HHT diagnosis.

The use of biomarkers in basic and clinical research, as well as in clinical practice, has become a commonplace in diagnosis, prognosis, and in primary endpoints of clinical trials and preclinical research studies (Vasan, 2006). The term biomarker refers to a broad subcategory of medical signs which can be measured accurately and reproducibly. In 1998, the National Institutes of Health (NIH) Biomarkers Definitions Working Group (2001) defined a biomarker as “a characteristic that is objectively measured and evaluated as an indicator of normal biological processes, pathogenic processes, or pharmacologic responses to a therapeutic intervention.” Over the last two decades, a large number of studies have searched for different potential biomarkers in HHT. Different ways have been developed in the past years to improve and facilitate the HHT molecular diagnosis. On one hand, advances in sequencing procedures have allowed quicker and more precise sequencing of several genes simultaneously. In this context, the recent design of new platforms for the capture and sequencing of a panel of genes involved in HHT and related diseases may change the sequencing approach. On the other hand, emerging non-sequencing methods based on soluble plasma markers, microRNAs (miRNAs; miRs), or differential physical patterns distinguishing HHT patients from the non-HHT population have been described. The pathogenic haploinsufficiency of the HHT gene products yields a deregulated genetic expression pattern in the cells most targeted in HHT, the endothelial cells (Fernandez-L et al., 2007a). As a result, an altered pattern of protein expression levels, including soluble products, could be detected in plasma using standard blood tests. In this same line, the detection of miRNAs related to HHT biology is also a useful tool which emerges to complete the diagnostic field.

### Direct Diagnostic Methods Based on Genetic Analysis: Sanger and Next Generation Sequencing

**FIGURE 2:**
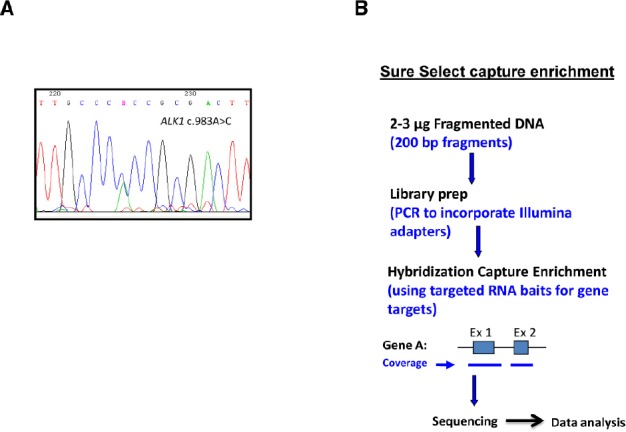
**Sanger sequencing vs next generation sequencing (NGS). (A)** Typical chromatogram showing the results of Sanger sequencing. **(B)** Sequencing scheme following capture enrichment of gene targets in a panel of diagnosis. NGS involves three major components: sample preparation, sequencing, and data analysis. The process begins with extraction of genomic DNA from a patient sample. Library generation is the process of creating random DNA fragments, of a certain size range, here represented by 200-bp, that contain adapter sequences on both ends. This library is the required input for most currently available NGS platforms. The adapters are complementary to platform specific PCR and sequencing primers. Target enrichment is achieved by RNA bait hybridization with the specific genes of interest. Adapted from Wooderchak-Donahue et al. (2012).

The classical Sanger sequencing analysis of genes, including genes found as the main cause for HHT, *ENG* and *ACVRL1/ALK1* and sometimes *MADH4/SMAD4* (Figures 2A,B) is currently the method followed in most genetic diagnosis laboratories, complemented with the MLPA (multiplex ligation-dependent probe amplification) technique for large deletion/insertions of exons. In addition, an array CNV/CGH (copy number variation/comparative genomic hybridization) to detect even larger duplications/deletions was also proposed to complement the previous panel (Fontalba et al., 2013).

However, ARUP Laboratories, Salt Lake City, UT, USA, recently proposed the use of a next generation sequencing (NGS) panel for a more efficient genetic analysis in the diagnosis of HHT and other vascular malformations (Figure 2B; McDonald et al., 2015). The idea is to design a panel including genes mutated in HHT and other vascular alterations with partial phenotype/genotype overlapping with HHT: HHT (*ENG*, *ALK1/ACVRL1*, *MADH4/SMAD4*, *BMP9/GDF2*), pulmonary arterial hypertension (*PAH*) (*BMPR2*, *CAV1*, *ALK1/ACVRL1*), cerebral cavernous malformation (CCM) (*KRIT1*, *CCM1*, *CCM2*, *PDCD10*), and capillary malformation-AVM (CM-AVM) syndrome, caused by mutations in *RASA1*. This way, the genetic screening includes not only *ALK1/ACVRL1* and *ENG*, but many more genes for almost the same cost. Currently, this new genetics platform is under a validation phase with clinical cases and controls. The cost of the whole analysis using the platform is only slightly superior to the traditional Sanger method. In summary, the design of a vascular malformation NGS panel is clinically useful, cost-effective, and can be updated annually to include additional vascular malformation genes, as they are identified. This approach has been recently applied to diseases affecting the joint such as aortopathies. Thus, a direct comparison of NGS enrichment methods using an aortopathy gene panel, show a clear improvement of the clinical diagnostics perspective (Wooderchak-Donahue et al., 2012).

### Plasma and Serum Proteins as Potential Biomarkers of HHT: TGF-β1, VEGF, Ang-2, and sEng

**FIGURE 3:**
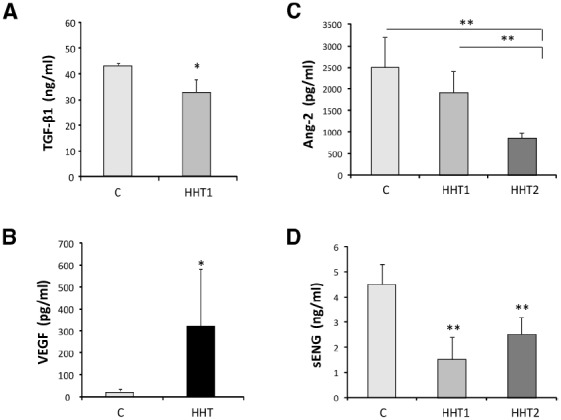
**Circulating protein biomarkers in HHT. (A)** The levels of TGF-β1 secreted (over 7 h) by 15 different HUVEC with HHT1 were significantly different (*p < 0.05) from the control HUVEC (C) tested in the same experiment. **(B)** VEGF plasma concentration levels in 25 healthy controls and 18 HHT patients. The mean values and the standard deviation are given for each group (*p < 0.001). **(C)** Plasma levels of Ang-2 and **(D)** plasma levels of sEng in healthy donors (*n* = 38), patients with HHT1 (*n* = 32) and patients with HHT2 (*n* = 30). Significant differences between groups (**p < 0.01) are shown. Adapted from Letarte et al. (2005) **(A)**, Sadick et al. (2005) **(B)**, and Ojeda-Fernandez et al. (2010) **(C,D)**.

Altered levels of several plasma/serum proteins in HHT patients and HHT animal models have been reported. Among these proteins are transforming growth factor β1 (TGF-β1), vascular endothelial growth factor (VEGF), angiopoietin 2 (Ang-2), and soluble endoglin (sEng), all involved in vascular biology, including angiogenesis and vascular remodeling (Figure 3).

#### Transforming Growth Factor β1

There are different genes mutated in HHT, *ENG*, *ACVRL1*, GDF2/BMP9, and *MADH4* whose proteins are involved in the TGF-β superfamily signaling pathway of vascular endothelial cells (Bernabeu et al., 2010). Based on this link several groups have measured the levels of TGF-β1 in HHT patients, as the prototypic member of the TGF-β family.

Thus, Sadick et al. (2005) reported increased plasma concentrations of TGF-β1 in German HHT patients with respect to the control population (50 ng/mL vs 6 ng/mL, respectively). By contrast, Letarte et al. (2005) found lower plasma levels of TGF-β1 in the Canadian HHT population compared to the control population (33 ng/mL vs 44 ng/mL, respectively; Figure 3A). Moreover, no differences in plasma TGF-β1 and -β2 levels of Chinese HHT2 patients were observed, compared to those of normal subjects (Zhang et al., 2004; Peng et al., 2006). The reasons for these discrepancies may be found in the different genetic background of the human populations involved. While the first study did not discriminate between HHT1 and HHT2 patients, Letarte et al. (2005) showed that HHT1 patients had the lowest levels of TGF-β1 (average 25.5 ng/mL), whereas the HHT2 population had a mean value not significantly different from control population (40 ng/mL in HHT2 vs 44 ng/mL). Because the German HHT population analyzed was predominantly HHT2 (Sadick et al., 2009) compared to the Canadian one, with more HHT1 patients present in the study, the differences in TGF-β1 levels may reflect geographical differences in the predominant HHT subtype of populations (Shovlin, 2010). These data and the marked differences in TGF-β1 levels detected in the control population (6 ng/mL vs 44 ng/mL) suggest a variety of factors which may influence an accurate measurement of TGF-β1, including sample collection, plasma vs serum, discrimination between latent and active forms of TGF-β, release of TGF-β1 from platelets, clinical heterogeneity, or lack of the standardized methodologies, leading to a limited interpretation of the results and currently argue against TGF-β1 levels as being a good biomarker of HHT.

TGF-β1 levels are subject to variability depending on the genetic background and may be reduced especially in HHT patients with higher penetrance of symptoms. Indeed, Bourdeau et al. (2001) showed that TGF-β1 circulating levels change significantly depending on the mouse strain. Thus, TGF-β1 was significantly lower in 129/Ola than in C57BL/6 strain. Interestingly, disease prevalence in endoglin heterozygous mice is much higher in 129/Ola (72%) than in C57BL/6 animals (7%) (Bourdeau et al., 1999). These data suggest that modifier genes regulating TGF-β1 expression act in combination with HHT genes in the development of the HHT phenotype. Thus, the levels of TGF-β1 may be variable and correlate with the severity of symptoms in HHT patients, depending on genetic modifiers. Therefore, TGF-β1 levels should not be considered as a real HHT biomarker, but rather a severity marker in the HHT genetic background.

#### Vascular Endothelial Growth Factor

Previous studies have proposed that haploinsufficiency of HHT genes impairs the TGF-β1 signaling pathway, leading to abnormal vascular remodeling and angiogenesis where factors, such as VEGF or Ang-2, play a potential role in HHT angiodysplasia (Bernabeu et al., 2010). VEGF, also known as vascular permeability factor, is a cytokine that induces proliferation and migration of endothelial cells to form new vessels and increases vascular permeability. Interestingly, anti-VEGF therapy has recently shown beneficial results in HHT patients (Dupuis-Girod et al., 2012). These benefits showed that systemic treatment with bevacizumab is promising only in symptomatic patients with organ involvement and life-threatening conditions (Kanellopoulou and Alexopoulou, 2013).

Early observations by Cirulli et al. (2003) indicated increased concentration of VEGF in the serum of HHT patients compared to control subjects (196.3 pg/mL vs 152.0 pg/mL, respectively), proposing VEGF as a possible diagnostic marker for HHT screening. These results were later confirmed by Sadick et al. (2005) who showed that plasma VEGF levels are 10-fold higher on average in a group of 31 HHT patients than in a comparable number of non-HHT population (331 pg/mL vs 20 pg/mL, respectively; Figure 3B). Similarly, significantly higher levels of VEGF were found in affected HHT2 family members as compared to normal and unaffected relatives (Peng et al., 2006). In summary, VEGF levels are increased in HHT patients compared to the control population. There is no difference in plasma (or tissue) VEGF levels between HHT1 and HHT2 patients (Sadick et al., 2008). These results suggest that continuous VEGF hyper-stimulation may lead to the development of abnormal micro-vessels due to unbalanced angiogenesis, as seen in HHT patients. In spite of the fact that the angiogenic factor VEGF is a putative player in the pathogenesis of HHT, it cannot serve as a specific diagnostic marker to discriminate between HHT1 and HHT2. Nonetheless, because most of these studies were carried out in adults, the existence of different VEGF levels between HHT1 and HHT2 in children cannot be excluded.

Higher levels of VEGF in plasma were shown to be related to higher epistaxis or gastric bleeding in a study by Giordano et al. (2009), and recently with a few cases in our laboratory (unpublished results). If these observations were confirmed in future studies and plasma VEGF levels were shown to be related to disease severity, one would not expect high VEGF levels in children, who generally exhibit sporadic or little bleeding. The role of VEGF in the generation of AVMs is inferred by the induction of abnormal microvasculature in the endoglin heterozygous mouse brain, after overexpression of VEGF (Xu et al., 2004). These findings may stress the importance of knowing VEGF levels, before starting an anti-angiogenic treatment in HHT population, specially, when using Avastin (anti-VEGF antibodies).

#### Angiopoietin-2

Angiopoietin-2 was considered as an HHT marker, following gene expression studies of microarrays (Fernandez-L et al., 2007a). In fact, Ang-2 was identified as a down-regulated gene in endothelial cells from HHT patients. Ang-2 is a family member of vascular growth factors, including angiopoietin-1 (Ang-1), that plays a role in embryonic and postnatal angiogenesis. Ang-1 is critical for vessel maturation, whereas Ang-2 works as an antagonist of Ang-1 and promotes vessel regression in the absence of VEGF. Thus, Ang-2 synergizes with VEGF to increase proliferation and migration of endothelial cells (Eklund and Saharinen, 2013).

Ojeda-Fernandez et al. (2010), quantified by enzyme-linked immunosorbent assay (ELISA) the levels of Ang-2 in plasma from HHT patients and controls. Levels of Ang-2 were reduced in HHT (HHT1, *n* = 32; and HHT2, *n* = 30) patients compared to the control population (*n* = 38; Figure 3C). However, some quantitative differences between the two main HHT types were observed. While there were no significant differences between HHT1 and healthy donors, significant differences between HHT2 and healthy subjects or HHT1 patients, were found.

#### Surface and Soluble Endoglin

Analysis of endoglin surface protein levels in affected patients strongly supports haploinsufficiency as the underlying cause of HHT1. A comprehensive review by Abdalla and Letarte (2006), on HHT1 samples analyzed over 10 years showed that endoglin levels on activated monocytes and endothelial cells [human umbilical vein endothelial cells (HUVECs)] of individuals with *ENG* mutations are reduced almost to 50% (Pece-Barbara et al., 1999; Abdalla and Letarte, 2006). Similarly, most *ACVRL1/ALK1* mutations lead to unstable and non-functional mutant proteins, supporting haploinsufficiency as the predominant model of HHT2 (Abdalla et al., 2000; Abdalla and Letarte, 2006). In HHT1, as recapitulated by Abdalla and Letarte (2006), the measure of decreased endoglin protein levels on the surface of macrophages and endothelial cells, is a good marker for HHT1. Furthermore, a decreased expression of endoglin and ALK1 on activated monocytes and blood outgrowth endothelial cells (BOECs) from HHT patients has been described (Sanz-Rodriguez et al., 2004; Fernandez-L et al., 2007a), as discussed below.

On the other hand, sEng has been associated with several cardiovascular pathologies such as preeclampsia, acute myocardial infarction, and tumor development (Li et al., 2000; Takahashi et al., 2001; Venkatesha et al., 2006; Cruz-Gonzalez et al., 2008; Hawinkels et al., 2010; Valbuena-Diez et al., 2012). Upregulated sEng levels are linked to poor cancer prognosis and correlate with metastases in patients with breast cancer. Furthermore, sEng levels in the sera of pregnant women increase and become strongly elevated, if women are developing preeclampsia, a pregnancy-specific hypertensive syndrome (Venkatesha et al., 2006). This soluble protein is thought to be generated by proteolytic cleavage of the membrane anchored endoglin, a TGF-β auxiliary receptor involved in angiogenesis and vascular remodeling and development (Hawinkels et al., 2010; Valbuena-Diez et al., 2012). Thus, sEng levels in HHT1 patients are expected to be lower compared with those of control subjects because HHT1 results from endoglin haploinsufficiency (Abdalla and Letarte, 2006). Moreover, endoglin deficiency is detected, to a lesser extent, in macrophages and BOECs from HHT2 patients (Sanz-Rodriguez et al., 2004; Fernandez-L et al., 2006). In HHT1, endoglin expression on the cell surface is reduced by approximately 50% (Sanz-Rodriguez et al., 2004; Fernandez-L et al., 2007a) while membrane endoglin levels in HHT2 range from 90 to 25%, depending on the type of *ACVRL1/ALK1* mutation, the age of the patient, and the disease severity (Fernandez-L et al., 2006, 2007a). These studies prompted Ojeda-Fernandez et al. (2010) to quantify by ELISA the levels of sEng, in 32 HHT1, 30 HHT2, and 38 control donors (Figure 3D). The levels of soluble endoglin were significantly different in the three groups, suggesting that sEng could be a biomarker not only for the HHT condition, but also to distinguish between HHT1 and HHT2. Based on the results of this study, a protocol for diagnosis was proposed combining the Ang-2 and sEng levels in a discriminant function analysis. Reference levels for Ang-2 and sEng indicative of HHT diagnosis with 95% cut-off values were: (i) non-HHT: sEng > 3.66 ng/mL and Ang2 > 2179.17 pg/mL; (ii) HHT1: sEng < 1.71 ng/mL, regardless Ang2 levels; and (iii) HHT2: sEng > 3.19 ng/mL and Ang2 < 1128.17 pg/mL.

In summary, down-regulated protein levels of Ang-2 and soluble endoglin in plasma represent potential HHT biomarkers in the biochemical diagnosis of HHT facilitating the rapid identification of suspected HHT patients. However, before using Ang-2 and sEng as a diagnostic test, specificity and sensitivity must be determined in large groups of patients.

### Prothrombotic Factors in HHT

HHT causes chronic nasal and gastrointestinal hemorrhage and prothrombotic agents are commonly used for severe hemorrhage. The use of antifibrinolytic agents, such as ε-aminocaproic or tranexamic acids, systemically administered using oral administration show satisfactory results with an improvement in epistaxis and the associated anemia (Fernandez-L et al., 2007b; Shovlin, 2010). However, the thrombotic risks of these drugs have not been well defined in HHT and they are contraindicated in those patients prone to thrombosis. For this reason, the screening for levels of coagulant factors VIII and V and von Willebrand factor in HHT has been proposed (Shovlin et al., 2007). These biomarkers of a prothrombotic state may be very useful before starting an antifibrinolytic treatment in HHT patients. In order to identify prothrombotic variables in HHT patients, and assess their potential functional significance, a pilot ELISA-based study comparing plasma proteins in healthy individuals with HHT to age/sex-matched non-HHT controls was validated in a full study of 309 consecutive HHT patients (Shovlin et al., 2007). In the pilot study, factor VIII and von Willebrand factor antigen concentrations were elevated in the HHT group compared to non-HHT controls. Therefore, the increased levels of factor VIII and other prothrombotic factors may influence thrombotic risk in HHT. These results suggest that in HHT patients prone to hypercoagulability, the therapies to avoid bleeding may lead to the risk of suffering deep venous thromboembolism. Accordingly, the measurement of these prothrombotic biomarkers may contribute to an individualized risk-benefit consideration and may be helpful in HHT management (Shovlin et al., 2007; Shovlin, 2010).

### MicroRNAs and Long Non-Coding RNAs as Biomarkers for HHT

MicroRNAs are short (±22 nucleotides long), non-coding RNAs that post-transcriptionally repress gene expression by targeting the 3′-untranslated regions (3′-UTR) of specific mRNAs (Bartel, 2009). Some miRNAs play a major role in vascular biology (Yang et al., 2005; Zampetaki and Mayr, 2012; Sayed et al., 2014) and the existence of the so-called “angio-miRs” (miRNAs that stimulate or repress angiogenesis) have opened up a novel aspect for therapeutics associated with deregulated angiogenesis and vascular diseases (Kane et al., 2014). Recently, the detection of specific miRNAs in plasma has emerged as a promising diagnostic tool and investigations are currently under development in many diseases.

**FIGURE 4:**
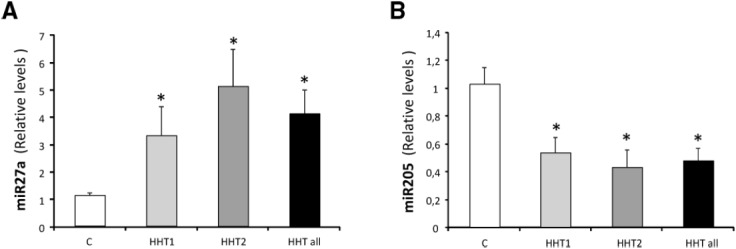
**miRNAs as HHT biomarkers in plasma.** Levels of **(A)** miR-27a and **(B)** miR-205 were measured in plasma samples of a total of 24 HHT patients HHT1 (*n* = 11) and HHT2 (*n* = 13) and in 16 healthy controls by qRT-PCR. Data are expressed as relative miRNA levels normalized to two spikes-in *C. elegans* miRNAs and are expressed as mean ± SEM. *p < 0.05 vs healthy patients. Adapted from Tabruyn et al. (2013).

Recently, Tabruyn et al. (2013) have demonstrated a circulating miRNA signature that could help to identify HHT patients. In this work, 24 HHT patients (11 HHT1 and 13 HHT2) and 16 healthy controls were included. While the levels of miR-27a were significantly higher in HHT patients than in controls, miR-205 was significantly downregulated in plasma from HHT patients (Figure 4). The miR-27 had been previously described as a pro-angiogenic micro-RNA (Zhou et al., 2011), but the role of miR-205 in endothelial cells was unknown. In this work, miR-205 was shown to reduce endothelial cell proliferation, migration, and tube formation. In addition, the authors showed that the expression of miR-205 modulates the TGF-β1 pathway, by targeting Smad1 and Smad4. It can be speculated that the combination of miR-205 and miR-27a could be potentially used in future to follow disease evolution, as well as to design antiangiogenic therapies based on restoring the normal levels of these altered miRs.

Zhang et al. (2013), in an independent study, reported the relationship between the miR-210 and the presence of pulmonary AVMs (PAVMs). These AVMs are present in 30–50% of patients with HHT and can lead to bleeding, stroke, and other complications. A total of eight circulating miRNAs were found altered in HHT patients with PAVMs. Among them, real-time RT-PCR showed that the levels of circulating miR-210 were significantly elevated in HHT patients with PAVMs, but remained unchanged in patients without PAVMs. Interestingly, miR-210 has been shown to be overexpressed under hypoxic conditions (Valera et al., 2011) and is present in sporadic renal carcinoma, and von Hippel–Lindau (VHL) dependent carcinoma (Wang et al., 2014). Therefore, the presence of increased miR-210 levels could be related to hypoxemia caused by the PAVMs. Further investigations in large patient groups and sensitivity studies are needed before circulating miR-210 could be considered as a biomarker for the screening of clinically significant PAVMs in patients diagnosed with HHT.

Torring et al. (2014) have recently described the first study to assess the regulatory effects of long non-coding RNAs (lncRNAs) in HHT affected tissue. lncRNAs are non-protein coding transcripts longer than 200 nucleotides that are involved in transcriptional, post-transcriptional and epigenetic regulation (Bonasio and Shiekhattar, 2014). Using microarray technology, Torring et al. (2014) identified lncRNAs that are differentially expressed in HHT telangiectasial tissue compared with HHT non-telangiectasial nasal tissue. Analysis of the lncRNA transcriptome link to biology using the GREAT software revealed several gene ontology processes to be affected, including blood vessel morphogenesis, blood vessel development, and vasculogenesis. Of note, a central group of telangiectasial genes dysregulated in HHT appears to be cis-regulated by differentially expressed lncRNAs. This group of genes primarily includes *CAV1*, *CCM2*, *FOXF1*, *FZD4*, *PRSS23*, *RASA1*, *SMO*, *TIPARP*, *ZFPM2*, and *ZMIZ1*. Mutations in some of these genes are known to cause vascular malformations, as in HHT. For example, mutations in *CCM2* cause cerebral cavernous malformation type 2, whereas mutations in *RASA1* lead to CM-AVM syndrome.

### Mid-Infrared Spectroscopy and Artificial Neural Network Patterns to Discriminate HHT Patients from Non-HHT Donors

A completely different approach to establish an HHT signature, was carried out by Lux et al. (2013), based on infrared (IR) spectroscopy. IR-spectroscopy of body fluids like blood plasma, serum, or urine has revealed disease-specific changes in spectral signatures, for example, in diabetes mellitus (Petrich et al., 2000), β-thalassemia (Liu et al., 2003), myocardial infarction, and heart failure (Haas et al., 2010). Interestingly, this IR method is not measuring changes of single proteins, but encompasses the overall metabolic changes caused by the disease, translated into specific “IR-spectra.” In the paper of Lux et al. (2013), the subject of analysis was the “metabolic change pattern” derived from peripheral blood plasma. IR-spectra were obtained by Fourier-transform mid-IR spectroscopy from blood plasma of 192 HHT patients and 191 healthy donors. Spectral data were mathematically processed and subsequently classified and analyzed by artificial neural network (ANN) methods and by visual analysis of scatter plots of the dominant principal components. The analyses showed that for HHT, a disease specific IR-spectrum exists, and is significantly different from that of the control group. According to the authors, with this method, HHT can be diagnosed with a sensitivity and specificity of 95%.

### Data Mining for Novel HHT Biomarkers

Any biological parameter related with a disease state may be considered a biomarker. In this sense, even differential patterns of maturation and circulation of bone marrow cell precursors can be associated with HHT. Recently, Massa et al. (2015), have published that in HHT patients, the population of blood circulating CD34^+^ endothelial cells is increased, irrespective of the disease variant (HHT1 or HHT2). In patients with an *ENG* mutation, the frequency of the cell subsets inversely correlated with the age of the patients at time of sampling (CD34^+^), disease duration (CD34^+^, VEGFR-2^+^), and age at disease onset (CD34^+^, CD133^+^, VEGFR-2^–^). Moreover, Zucco et al. (2014) reported that the percentage of CD34^+^ cells in peripheral blood mononuclear cells from HHT patients was significantly higher than in controls.**** Interestingly, the CD34^+^ cell subset has been involved in the repair of damaged vessels (Yoder, 2012) and its increased numbers could give some hints regarding the severity of the vascular lesions in HHT. It will be interesting to investigate whether other the blood cell subsets are also altered in HHT.

HHT biomarkers are expected to be the consequence of the deregulated expression of HHT genes. Within the last years, several omics analysis from either over- or under-expressed endoglin or ALK1 cellular systems have led to considerable information related to the target genes/proteins modulated by these HHT genes. Among them: expression microarrays of the downstream target genes affected by haploinsufficiency in endoglin or ALK1 in HHT human endothelial cells from patients. These studies identified 168 genes downregulated, and only, 40 upregulated genes. Differentially expressed genes are involved in migration, angiogenesis, cell guidance, cytoskeleton organization, intercellular connections, cell proliferation, or nitric oxide (NO) synthesis (Fernandez-L et al., 2007a).

Another microarray study was performed with endothelial cells from HHT and control umbilical cords (Thomas et al., 2007). Results revealed that HHT endothelial cells had differentially expressed genes associated with the angiogenesis activation phase, cell guidance, intercellular connections, and with the TGF-β signaling pathway.

Lux et al. (2006), using a different approach, infected a human microvascular cell line with a recombinant constitutively active ALK1 adenovirus and studied gene expression. Gene array analysis identified 49 genes to be regulated by ALK1 signaling, including at least 14 genes involved in angiogenesis.

Recently, expression microarrays and proteomic analysis of human monocytic cells constitutively overexpressing endoglin has shown hundreds of deregulated proteins involved in cellular activities affected during aging, as well as essential biological functions, mainly those related to cellular movement, including cell adhesion and transmigration (Aristorena et al., 2014; Blanco et al., 2015) These transcriptome- and proteome-wide studies have only scratched the surface of the great deal of HHT related gene/protein data. Hopefully, the published and upcoming data obtained from all these experiments may constitute a valuable source of information that could be used in the future to identify novel HHT biomarkers.

## Author Contributions

LMB contributed to the conception, acquisition of material, drafting of the work, revising the manuscript critically, ensuring accuracy and integrity of the different views in each section, and providing own experience in the field. VA contributed to the design of the work, analysis of it, revising critically the manuscript final approval. LO-F contributed to the analysis of the work, co-author of some of the results shown, revision of the manuscript, support in intellectual content. LR-P, helped with some of the techniques whose results are reviewed and shown, the acquisition and analysis of the results, final approval of the version to be published, and assistance in the revision and editing of the manuscript. CB made substantial contributions to the conception of the work, interpretation of the work, drafting and revising critically the manuscript for important intellectual content, agreement to be accountable for all aspects of the work in ensuring that questions related to the accuracy and integrity of the work are appropriately investigated.

### Conflict of Interest Statement

The authors declare that the research was conducted in the absence of any commercial or financial relationships that could be construed as a potential conflict of interest.
